# Outcomes After Transcatheter Mitral Valve Replacement in Valve in Valve, Valve in Ring, and Mitral Annular Calcification

**DOI:** 10.1016/j.jscai.2025.104003

**Published:** 2025-11-18

**Authors:** Chikashi Nakai, Augustin DeLago, Sanjay Samy

**Affiliations:** a Department of Cardiothoracic Surgery, Albany Medical Center, Albany, New York; b Department of Cardiology, Capital Cardiology Associates, Albany, New York

**Keywords:** transcatheter mitral valve replacement, valve in mitral annular calcification, valve in ring, valve in valve

## Abstract

**Background:**

There are few reports about midterm to long-term outcomes of transcatheter mitral valve replacement (TMVR) in valve in valve (ViV), valve in ring (ViR), and valve in mitral annular calcification (ViM). The aim of this study was to assess postoperative outcomes in patients who underwent TMVR for calcification or failing valves.

**Methods:**

Between March 2016 and July 2024, 82 patients underwent TMVR with SAPIEN 3 (Edwards Lifescience) balloon-expandable valve, with 72 transseptal and 10 transapical access. Of 82 patients, 47 had TMVR with ViV/ViR and 35 with ViM. Postprocedural outcomes and midterm survivals were evaluated comparing ViV/ViR group with ViM group.

**Results:**

The ViM required more ventricular septal ablation before TMVR for left ventricular outflow tract obstruction (31.4% [11/35] vs 4.3% [2/47]; *P* < .01). Early hospital mortality was significantly higher in the ViM group (34.5% [12/35] vs 2.1% [1/47]; *P* < .01). The incidence of postprocedural stroke, left ventricular outflow tract obstruction, permanent pacemaker, and reintervention for mitral valve was similar between the 2 groups. The mean follow-up term was 17.6 ± 22.5 months. The cumulative 5-year survivals in ViV/ViR and ViM were 57.4% and 24.3%, respectively. A significant difference was noted in the midterm outcome between the 2 groups (*P* < .01). On multivariable Cox proportional hazards analysis, only conducting ViM procedure was associated with an increased midterm mortality (hazard ratio, 2.58; 95% CI, 1.33-4.99; *P* < .01).

**Conclusions:**

Short-term and midterm outcomes of TMVR in ViV and ViR were better than those of ViM in this patient cohort.

## Introduction

Mitral annular calcification increases the risk of stroke, cardiovascular disease, and death.^1^ However, surgical mitral valve replacement in this population is technically challenging for calcium burden.^2^ Redo mitral valve surgery for degenerated bioprosthetic valve or ring is associated with high mortality and morbidities in elderly patients.^3^ In this context, transcatheter mitral valve replacement (TMVR) has been used for patients with failing bioprosthetic valves and rings or severe mitral annular calcification who were considered as high risk for open-heart surgery.^4^ Recent report from the MITRAL (Implantation of Transcatheter Valves (MITRAL) Trial demonstrated improvement of mortality in patients who underwent valve in valve (ViV) and valve in ring (ViR) TMVR. However, mortality in patients who received valve in mitral annular calcification (ViM) TMVR remains poor.^5^ On the basis of these findings, patients in this study were divided into 2 groups, ViV/ViR and ViM, to elucidate outcomes, including the midterm survival.

## Materials and methods

This study included consecutive patients who underwent TMVR with a SAPIEN 3 (Edwards Lifesciences) balloon-expandable valve (BEV) at Albany Medical Center between March 2016 and July 2024. All patients were evaluated by the Albany Medical Center structural heart team, and TMVR was indicated for patients with severe mitral regurgitation (MR) or mitral stenosis (MS) and New York Heart Association (NYHA) functional class II or greater who were considered as high risk for open-heart surgery. In total, 82 patients were identified for this study; 47 patients had ViV/ViR TMVR and 35 ViM TMVR. Of the 47 patients in the ViV/ViR group, 28 underwent ViV procedure and 19 ViR procedure. Our institutional review board approved the protocol (No. 7111; December 2, 2024), and the need for individual informed consent was waived. We conducted a chart review on all patients in the electronic health records system at Albany Medical Center. Preoperative, intraprocedural, and postoperative data were collected in a dedicated database prospectively. All postoperative patients were followed up in 30 days, 1 year, and yearly thereafter.

### TMVR procedure

#### Transseptal approach

An access to the right femoral vein was obtained and a 5F sheath was placed. A wire was placed into the inferior venous cava and a 16F sheath was advanced over the wire. Under transesophageal echocardiogram guidance, transseptal puncture was performed, and a wire was advanced to the left atrium first and then advanced to the left ventricle with a steerable sheath. The interatrial septum was dilated with a 14-mm balloon. A SAPIEN 3 BEV was delivered into the mitral position across the atrial septum. Patients’ hearts were rapidly paced, and the valve was deployed.

#### Transapical approach

A 4-cm incision was made in the fifth intercostal space after localized with transthoracic echocardiogram. The intercostal space was entered, and finger palpitation performed to make sure the position of the apex of left ventricle. A minimally invasive chest retractor was placed to spread the intercostal space. The pericardium was opened after the position of apex was identified with the echocardiogram guidance. Polypropylene sutures were placed in 2 layers in a purse string fashion around the apex of left ventricle, and the access to the apex was obtained. Under fluoroscopic and transesophageal echocardiogram guidance, a wire was placed in the left atrium. Over the wire, an 18F sheath was placed into the left ventricle. A SAPIEN 3 BEV was delivered to the mitral position through the sheath. Patients’ hearts were rapidly paced, and the valve was deployed.

### Study outcomes

Early hospital mortality was defined by the death within 30 days after TMVR procedure. The primary end point was early hospital mortality and midterm survival in the ViV/ViR and ViM group. The secondary end point was NYHA functional classification and findings of echocardiogram at postoperative 30 days and 1, 3, and 5 years. As subanalyses, the midterm survivals were assessed in patients with high Society of Thoracic Surgeons (STS) score. An STS score >8 was considered as high risk for open-heart surgery. In addition, mid-term survival between the ViV and ViR groups were evaluated.

### Statistical analyses

Categorical variables were described as numbers and percentages, while continuous variables were presented as mean ± SD. Student *t* test was performed for continuous variables that had a normal distribution, and Mann–Whitney *U* test was used for those that did not have a normal distribution. Categorical variables were assessed by the χ^2^ test and Fisher exact test. A value of *P* of <.05 was considered statistically significant. On univariable analyses, the predictors of early mortality were determined. The predictors of *P* < .15 in univariable analyses were selected for a logistic regression analysis. The model fitting was confirmed by the area under the receiver-operating characteristic curve and Hosmer–Lemeshow test. Kaplan-Meier method was carried out to evaluate the cumulative survival, and statistical differences were analyzed using a log-rank test. Cox proportional hazard analysis was conducted to evaluate the predictors of midterm mortality. Three predictors (age, STS score, and ViM TMVR procedure) of midterm mortality were chosen. The proportional hazard assumption was verified by stratified log minus log plots and independent of time in covariables. The severity of MR and NYHA functional classification at each follow-up were compared using a paired Wilcoxon test. There were no missing data. Data analyses were performed by SPSS version 29.0.0.0 (IBM Corp) and Prism version 10.4.1 (GraphPad Software).

## Results

Baseline characteristics are listed in Table 1 and intraoperative and postoperative data in Table 2. Briefly, the mean age was 75.5 ± 10.7 years. A higher prevalence of men (51.1% vs 25.7%; *P* = .02) was observed in the ViV/ViR group, while the number of patients with diabetes (45.7% vs 19.1%; *P* < .01) and severe MS (85.7% vs 53.2%; *P* < .01) was larger in the ViM group. Preoperative left ventricular ejection fraction reduced significantly in the ViM group (47.9% ± 11.7% vs 54.7% ± 4.8%; *P* < .01). In the ViV/ViR group, 47 patients (100%) received previous surgical mitral valve interventions, whereas 13 (37.1%) in the ViM group had underwent previous open-heart surgery without mitral valve intervention. Of the 47 patients, 28 (59.6%) underwent mitral valve replacement and 19 (40.4%) mitral valve repair. The mean STS score was 9.4 ± 7.1, and rate of STS score of ≥8 was not different between the 2 groups (*P* = .07). Alcohol septal ablation was undertaken as elective procedure 3 to 4 weeks before TMVR procedure in 2 patients (4.3%) in the ViV/ViR group and 11 (31.4%) in the ViM group. Alcohol septal ablation to reduce the risk of left ventricular outflow tract (LVOT) obstruction was significantly higher in the ViM group (*P* < .01).

**Table 1:** Baseline characteristics

Characteristic	All (N = 82)	ViV/ViR (n = 47)	ViM (n = 35)	*P*
Age, y	75.5 ± 10.7	73.8 ± 11.4	77.8 ± 9.2	.09
Male sex	35 (42.7)	24 (51.1)	9 (25.7)	.02
Hypertension	57 (69.5)	30 (63.8)	27 (77.1)	.20
Diabetes	25 (30.5)	9 (19.1)	16 (45.7)	<.01
Atrial fibrillation	42 (51.2)	28 (59.6)	14 (40.0)	.08
Hemodialysis	9 (11.0)	5 (10.6)	4 (11.4)	.59
Coronary artery disease	28 (34.1)	14 (29.8)	14 (40.0)	.33
Peripheral vascular disease	18 (22.0)	11 (23.4)	7 (20.0)	.71
Previous open-heart surgery	60 (73.2)	47 (100)	13 (37.1)	<.01
Previous mitral valve replacement	28 (34.1)	28 (59.6)	0 (0)	<.01
Previous mitral repair	19 (23.2)	19 (40.4)	0 (0)	<.01
NYHA functional class				
I	0	0	0	
II	15 (18.3)	3 (6.4)	12 (34.3)	
III	56 (68.3)	37 (78.7)	19 (54.3)	
IV	11 (13.4)	7 (14.9)	4 (11.4)	
Left ventricular ejection fraction, %	50.8 ± 10.0	47.9 ± 11.7	54.7 ± 4.8	<.01
Severe mitral regurgitation	29 (35.4)	20 (42.6)	9 (25.7)	.11
Severe mitral stenosis	55 (67.1)	25 (53.2)	30 (85.7)	<.01
Mean pressure gradient, mm Hg	11.8 ± 5.7	12.2 ± 6.0	11.4 ± 5.2	.53
Mitral valve area, cm^2^	1.1 ± 0.4	1.1 ± 0.4	1.0 ± 0.4	.57
STS score	9.4 ± 7.1	8.5 ± 7.4	10.6 ± 6.5	.20
<8	43 (52.4)	29 (61.7)	14 (40.0)	.07
≥8	39 (47.6)	18 (38.3)	21 (60.0)	.07
Septal ablation before TMVR procedure	13 (15.6)	2 (4.3)	11 (31.4)	<.01
Permanent pacemaker placement after septal ablation	2 (2.4)	1 (2.1)	1 (2.9)	.67

Values are mean ± SD or n (%).

NYHA, New York Heart Association; STS, Society of Thoracic Surgeons; TMVR, transcatheter mitral valve replacement; ViM, valve in mitral annular calcification; ViR, valve in ring; ViV, valve in valve.

**Table 2:** Procedural characteristics and in-hospital outcomes

Characteristic	All	ViV/ViR	ViM	*P*
Successful TMVR procedure	76 (92.7)	45 (95.7)	32 (91.4)	.65
BEV (SAPIEN 3)	82 (100)	47 (100)	35 (100)	>.99
23 mm	7 (8.5)	6 (12.8)	1 (2.9)	
26 mm	45 (54.9)	29 (61.7)	16 (45.7)	
29 mm	30 (36.6)	12 (25.5)	18 (51.4)	
Transseptal approach	75 (91.5)	41 (87.2)	34 (97.1)	.11
Transapical approach	7 (8.5)	6 (12.8)	1 (2.9)	.11
Improper position of first valve	13 (15.9)	6 (12.8)	7 (20.0)	.38
Additional valve deployment	12 (14.6)	8 (17.0)	4 (11.4)	.75
Residual perivalvular leak mild or greater	11 (13.4)	3 (6.4)	8 (22.9)	.03
Left ventricular outflow tract obstruction	3 (3.7)	1 (2.1)	2 (5.7)	.39
Embolized valve	3 (3.7)	1 (2.1)	2 (5.7)	.39
Emergent surgical intervention for mitral valve	2 (2.4)	0	2 (5.7)	.18
Cardiac arrest	2 (2.4)	0	2 (5.7)	.18
In-hospital outcomes				
Stroke	4 (4.9)	2 (4.3)	2 (5.7)	.57
Permanent pacemaker implantation	0	0	0	—
New onset of atrial fibrillation	6 (7.3)	3 (6.4)	3 (8.6)	.51
New dialysis	4 (4.9)	1 (2.1)	3 (8.6)	.31
Cardiogenic shock	11 (13.4)	4 (8.5)	7 (20.0)	.19
Cardiac arrest	3 (3.7)	0	3 (8.6)	.07
Vascular complication, major	3 (3.7)	2 (4.3)	1 (2.9)	.61
Vascular surgery intervention	2 (2.4)	2 (4.3)	0	.50
Mitral valve reintervention	2 (2.4)	0	2 (5.7)	.18
Early hospital mortality	13 (15.9)	1 (2.1)	12 (34.5)	<.01
Cardiac	11 (13.4)	1 (2.1)	10 (28.6)	<.01
Noncardiac	2 (2.4)	0	2 (5.7)	.18
Length of hospital stay, d	5.9 ± 7.8	6.6 ± 8.7	4.9 ± 6.2	.31

Values are mean ± SD or n (%).

BEV, balloon-expandable valve; TMVR, transcatheter mitral valve replacement; ViM, valve in mitral annular calcification; ViR, valve in ring; ViV, valve in valve.

### Procedural and in-hospital outcomes

Operative procedures did not differ between the 2 groups (Table 2), including procedure successful rate, incidence of embolized valve, and LVOT obstruction after TMVR. Two patients in the ViM group were complicated by intraoperative cardiac arrest secondary to cardiac tamponade and unsuccessful deployment, respectively. The incidence of residual perivalvular leak mild or greater was significantly higher in the ViM group (22.9% vs 6.4%; *P* = .03). Patients in the ViV/ViR group stayed at hospital for 6.6 ± 8.7 days and those in the ViM group (4.9 ± 6.2 days; *P* = .31). A significant difference was not detected in the incidence of postoperative stroke, new onset of atrial fibrillation, dialysis requirement, cardiogenic shock, vascular complication, and mitral valve reintervention. Among the ViV/ViR group, 1 patient died within 30 days from the procedure due to heart failure, and 12 in the ViM group because of 5 heart failure, 3 ventricular fibrillation/pulseless electrical activity, 1 cardiac tamponade, 1 LVOT obstruction, and 2 noncardiac. When compared with the ViV/ViR group, the early hospital mortality was significantly higher in the ViM group (34.5% vs 2.1%; *P* < .01) (Table 2). The logistic regression analysis indicated that the high STS score (odds ratio [OR], 1.13; 95% CI, 1.01-1.25; *P* = .03) and TMVR procedure for ViM (OR, 20.76; 95% CI, 2.11-204.05; *P* < .01) raised the early mortality (Table 3).

**Table 3:** Univariable and multivariable analyses for the early mortality in patients who underwent TMVR

Risk factors	Adjusted odds ratio	95% CI	*P*
Univariable analysis			
Age	1.04	0.98-1.11	.24
STS score	1.08	1.00-1.16	.04
Septal ablation prior to TMVR	0.21	0.06-0.80	.02
TMVR procedure for ViM	24.0	2.94-196.09	<.01
Logistic regression analysis			
STS score	1.13	1.01-1.25	.03
Septal ablation prior to TMVR	3.78	0.71-20.24	.12
TMVR procedure for ViM	20.76	2.11-204.05	<.01

STS, Society of Thoracic Surgeons; TMVR, transcatheter mitral valve replacement; ViM, valve in mitral annular calcification; ViR, valve in ring; ViV, valve in valve.

### Midterm outcomes

**Figure 1:**
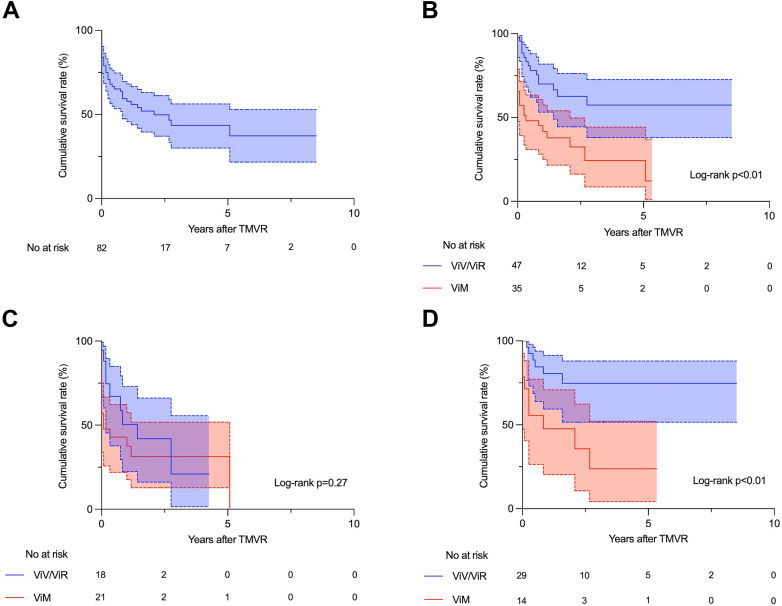
**Kaplan-Meier survival curves.** (**A**) Patients who underwent TMVR. (**B**) Patients who underwent ViV/ViR and ViM TMVR. (**C**) Patients who underwent ViV/ViR and ViM TMVR with an STS score of ≥8. (**D**) Patients who underwent ViV/ViR and ViM TMVR with an STS score of <8. STS, Society of Thoracic Surgeons; TMVR, transcatheter mitral valve replacement; ViM, valve in mitral annular calcification; ViR, valve in ring; ViV, valve in valve.

Clinical outcomes for 5 years of follow-up were described in Table 4. The mean follow-up period was 17.6 ± 22.5 months (Table 4). During these periods, 14 patients in the ViV/ViR group died (cardiac in 5 patients, noncardiac in 7 patients, and undetermined in 2 patients), along with 12 in the ViM group (cardiac in 6 patients, noncardiac in 4 patients, and undetermined in 2 patients). One patient was complicated by endocarditis in the ViV/ViR group, subsequently required the explantation of TMVR valve and surgical mitral valve replacement with mechanical valve. Three patients (2 in ViV/ViR and 1 in ViM) presented with partial LVOT obstruction; however, all patients were managed medically. The Kaplan-Meier survival curve in patients who underwent TMVR was described in Figure 1A. The survival rate in 5 years was 57.4% in the ViV/ViR group and 24.3% in the ViM group, respectively (Table 4). Figure 1B demonstrated a significant difference in the midterm survival rates between the 2 groups (*P* < .01) (Central Illustration, A). The cox proportional hazards regression analysis revealed that conducting TMVR for patients with ViM was an only significant predictor for an increased midterm mortality rate (HR, 2.58; CI, 1.33-4.99; *P* < .01) (Table 5) while age and STS score were not associated with a midterm mortality (Table 4). The Kaplan-Meier estimates of midterm survival rates in patients with STS score of ≥8 and <8 were shown in Figure 1C, D. The cumulative survival rates of STS score of ≥8 group were similar between the 2 groups (*P* = .27) (Figure 1C), whereas there was a significant difference in patients with STS score <8 (*P* < .01) (Figure 1D).

**Table 4:** Clinical events at 5 years

Event	All	ViV/ViR	ViM	*P*
Mean follow-up period, mo	17.6 ± 22.5	21.3 ± 25.0	12.5 ± 17.6	.07
Cumulative survival rate	43.5%	57.4%	24.3%	<.01
All-cause mortality	26 (31.7)	14 (29.8)	12 (34.3)	.67
Cardiac	9 (11.0)	5 (10.6)	6 (17.1)	.84
Noncardiac	11 (13.4)	7 (14.9)	4 (11.4)	.75
Undetermined	4 (4.9)	2 (4.3)	2 (5.7)	.57
Stroke	5 (6.1)	4 (8.5)	1 (2.9)	.39
Left ventricular outflow tract obstruction	3 (3.7)	2 (4.3)	1 (2.9)	.61
Endocarditis	1 (1.2)	1 (2.1)	0	.57
Surgical intervention for mitral valve	1 (1.2)	1 (2.1)	0	.57

Values are mean ± SD or n (%) unless noted.

ViM, valve in mitral annular calcification; ViR, valve in ring; ViV, valve in valve.

**Central Illustration:**
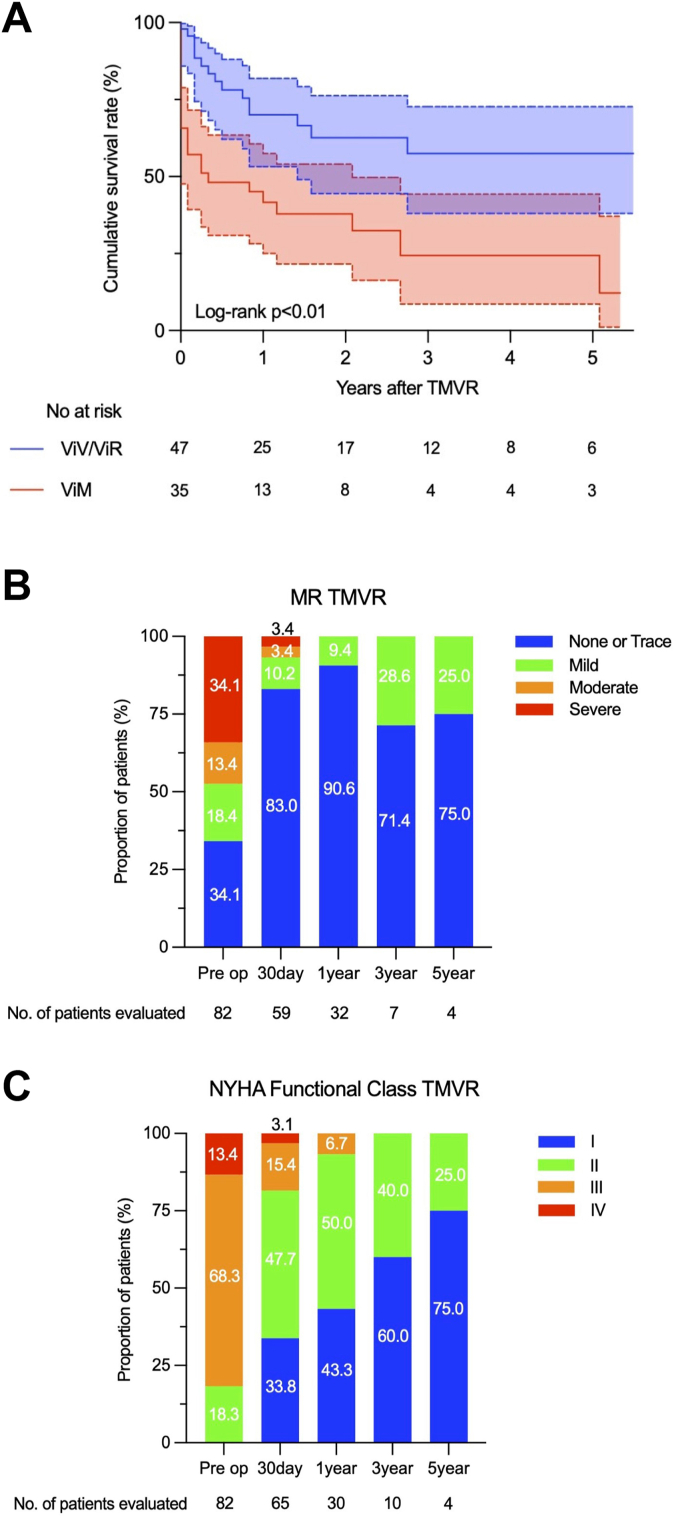
**Post Operative Outcomes After TMVR.** (**A**) Kaplan-Meier survival curves of ViV/ViR vs ViM TMVR. There was a significant difference in the midterm survival between the 2 groups (*P* < .01). (**B**) Severity of MR in patients who underwent TMVR: preoperative MR compared with postoperative MR. All postoperative patients presented with mild MR or less at 1-year follow-up, and severity reduced significantly. (**C**) NYHA functional classification in patients who underwent TMVR. Most patients at 1-year follow-up showed significant improvement of NYHA functional classification compared with their preoperative baselines. MR, mitral regurgitation; NYHA, New York Heart Association; TMVR, transcatheter mitral valve replacement; ViM, valve in mitral annular calcification; ViR, valve in ring; ViV, valve in valve.

**Table 5:** Multivariable analysis for midterm mortality in patients who underwent TMVR

Risk factors	Adjusted hazard ratio	95% CI	*P*
Age	1.01	0.98-1.05	.44
STS score	1.03	0.98-1.08	.15
TMVR procedure for ViM	2.58	1.33-4.99	<.01

TMVR, transcatheter mitral valve replacement; ViM, valve in mitral annular calcification.

### Severity of MR

**Figure 2:**
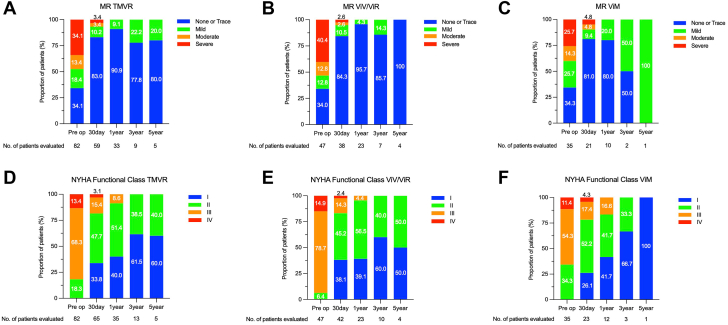
**Cumulative Survival Rates in Patients With TMVR.** (**A**) Severity of MR in patients who underwent TMVR. (**B**) Severity of MR in patients who underwent ViV/ViR TMVR. (**C**) Severity of MR in patients who underwent ViM TMVR. (**D**) NYHA functional classification in patients who underwent TMVR. (**E**) NYHA functional classification in patients who underwent ViV/ViR TMVR. (**F**) NYHA functional classification at preoperative and follow-up visits in patient who underwent ViM TMVR. MR, mitral regurgitation; NYHA, New York Heart Association; TMVR, transcatheter mitral valve replacement; ViM, valve in mitral annular calcification; ViR, valve in ring; ViV, valve in valve.

All patients who underwent TMVR presented with mild MR or less at the 1-year follow-up, and the severity of MR improved significantly when compared with the preoperative severity of MR (*P* < .01) (Figure 2A; Central Illustration, B). Over the follow-up period, postoperative MR tended to be ameliorated in both ViV/ViR and ViV groups (Figure 2B, C).

### NYHA functional classification

Most survivors at the 1-year follow-up described the significant improvement of NYHA functional classification compared with the preoperative baseline, 14 patients with class I, class II in 18, and class III in 3 (*P* < .01) (Figure 2D; Central Illustration, C). In the subanalysis of the ViV/ViR and ViV groups, the similar improvement was detected (Figure 2E, F).

### ViV vs VIR

**Figure 3:**
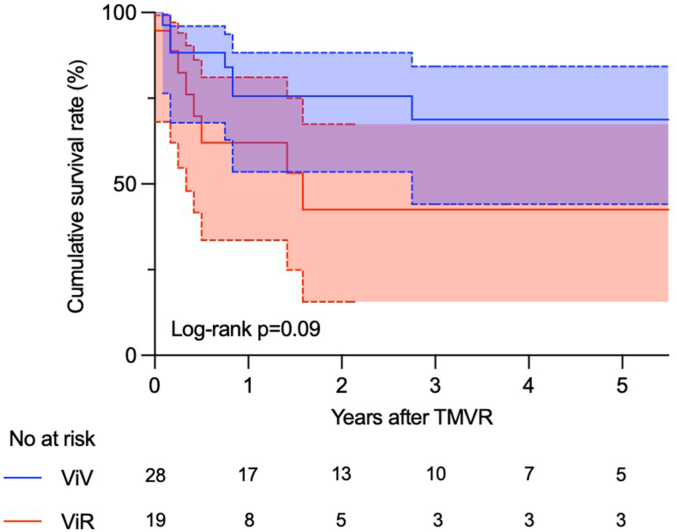
**Kaplan-Meier survival curves in patients who underwent ViV and ViR TMVR.** TMVR, transcatheter mitral valve replacement; ViR, valve in ring; ViV, valve in valve.

Preoperative characteristics, operative procedures, and postoperative outcomes were summarized in Supplemental Table S1. Age, sex, STS score, length of hospital stay, and the early mortality were similar between the 2 groups. The cumulative survival at 5 years was 68.8% in the ViV group and 42.5% in the ViR group (*P* = .09) (Figure 3).

## Discussion

The study findings are as follows: (1) patients who underwent ViV/ViR TMVR showed an acceptable cumulative survival in 5 years although a deteriorated midterm survival was observed in patients with ViM (57.4% vs 24.3%). (2) Among patients with STS score of <8, the midterm survival of patients who underwent ViV/ViR compared favorably with that of ViM. (3) Midterm outcomes between patients receiving ViV and ViR were similar. (4) Conducting TMVR procedure for mitral annular calcification was a significant predictor for an increased midterm mortality. (5) Survivors after TMVR procedure presented with improved NYHA functional class and the severity of MR.

Left ventricular outflow tract obstruction is one of critical complications related to the TMVR procedure caused by the displacement of the anterior leaflet covering the stent of the SAPIEN 3, which protrudes in the LVOT.^6^ The incidence varies between 7% and 39%.^4^ Preemptive alcohol septal ablation therapy has been investigated and shown promising results with an increase in neo-LVOT to avoid the LVOT obstruction.^7^ A high rate of permanent pacemaker implantation secondary to the septal ablation was reported, ranging from 16% to 35%.^7^^,^^8^ In our study, 13 patients (15.8%) underwent alcohol septal ablation. Of the 13 patients, 2 (15.4%) required permanent pacemaker for heart block, which was in agreement with the previous reports.

Several studies have recently addressed the 30-day mortality of ViV, ViR, and ViM groups (3.3%-8.1%, 6.7%-11.5%, and 11.1%-34.5%, respectively).^5^^,^^9^, ^10^, ^11^, ^12^ In our study, the early mortality rates in the ViV (0%) and ViR (5.3%) groups were better than those previously reported. The differences are thought to be multifactorial, including patient selection, improved operator experience, and measurements to decrease the risk of LVOT obstruction. However, the mortality in the ViM group (34.5%) was consistent with the previously reported findings. This could be explained by the fact that the prevalence of severe MS was more frequent in the ViM group and the procedure of ViM TMVR was still technically challenging when compared with other groups. Based on 5-year outcomes from the MITRAL Trial, 5year survival of ViV 79%, ViR 36%, and ViM 33%.^13^ Those results were almost similar to ours as follows: (1) ViV, 68.8%; (2) ViR, 42.5%; (3) and ViM, 24.3%. The ViM TMVR procedure was the only predictor for an increased long-term mortality in our cohort (hazard ratio, 2.58; *P* < .01). Midterm outcomes of the ViM group as well as short-terms outcomes remain poor due to complex comorbidities and procedures. The STS score has been used as a tool to assess surgical risks for mortality and comorbidities^14^ although the correlation between STS scores and outcomes regarding the TMVR procedure is poorly documented. Our study differs from others in that we compared the high STS score group with the intermediate-score group. The STS score in our study predicted the short-term mortality in the logistic regression analysis (OR, 1.13; *P* = .03) but was not a significant factor for the midterm outcome in the Cox hazard proportional analysis. The Kaplan-Meier estimates in patients with high STS score have shown no significant difference in the midterm survivals between the ViV/ViR and ViM groups (*P* = .27) (Figure 1C). Of particular note is that the patient’s prognosis might be poor regardless the type of the procedure. However, further study is needed to elucidate the relationship between STS scores and postoperative outcomes.

Several studies reported that there were improvements in NYHA functional class and postoperative MR.^13^^,^^15^ It must be emphasized that NYHA functional classification and the severity of MR in our cohort improved significantly from the preoperative baseline in all groups at each follow-up (Figure 2). Taken together, these results indicated the TMVR procedure might be worth to proceed to relieve symptoms.

This study include several limitations. First, it was a retrospective single-center study. A multicenter study is needed in the future because the surgical management might differ in each facility. Second, some patients were dead or lost during the follow-up periods. As a result, the number of remained patients decreased and the analyses at the 5-year follow-up demonstrated less powered. Third, there were confounders in this study populations including indication for the procedure (MR or MS), risk difference of LVOT obstruction in each group, and baseline comorbidities.

## Conclusion

Short-term and midterm outcomes of TMVR in ViV and ViR were better than that of valve in mitral annular calcification. ViM procedure was a predictor for an increased midterm mortality in this patient cohort. The NYHA functional classification and severity of MR were significantly improved after the procedure.
