# Supplementary material for: Outcomes After Transcatheter Mitral Valve Replacement in Valve in Valve, Valve in Ring, and Mitral Annular Calcification
Source: J Soc Cardiovasc Angiogr Interv. 2025 Nov 18;4(12):104003. doi: 10.1016/j.jscai.2025.104003 (PMC12766036; doi:10.1016/j.jscai.2025.104003)
Supplement: Supplemental Table S1 [file mmc1.docx]

**Supplemental Table 1 Preoperative, intraprocedural characteristics and postoperative outcomes in patients undergoing ViV and ViR**

| Characteristic | ViV n=28 | ViR n=19 | *p* value |
| --- | --- | --- | --- |
| Age, y | 75.0 ± 12.2 | 70.9 ± 10.0 | 0.16 |
| Male | 14 (50.0) | 10 (52.6) | 0.86 |
| STS score | 8.94 ± 7.54 | 7.95 ± 7.10 | 0.66 |
| Operative procedures |  |  |  |
| Successful procedure | 27 (96.4) | 18 (94.7) | 0.65 |
| Improper position of first valve | 2 (7.1) | 4 (21.0) | 0.20 |
| Additional valve deployment | 2 (7.1) | 5 (26.3) | 0.10 |
| Perivalvular leak mild or greater | 1 (3.6) | 2 (10.5) | 0.56 |
| Postoperative outcomes |  |  |  |
| Early hospital mortality | 0 (0) | 1 (5.3) | 0.40 |
| Length of hospital stay, day | 7.9 ± 9.8 | 4.8 ± 6.4 | 0.43 |
| Mean follow-up periods, month | 26.3 ± 28.1 | 12.8 ± 16.0 | 0.03 |
| Cumulative survival rate in 5years, % | 68.8 | 42.5 | 0.09 |

Values are mean ± SD or n (%)

STS = The Society of Thoracic Surgeons, ViR = valve in ring, ViV = valve in valve
